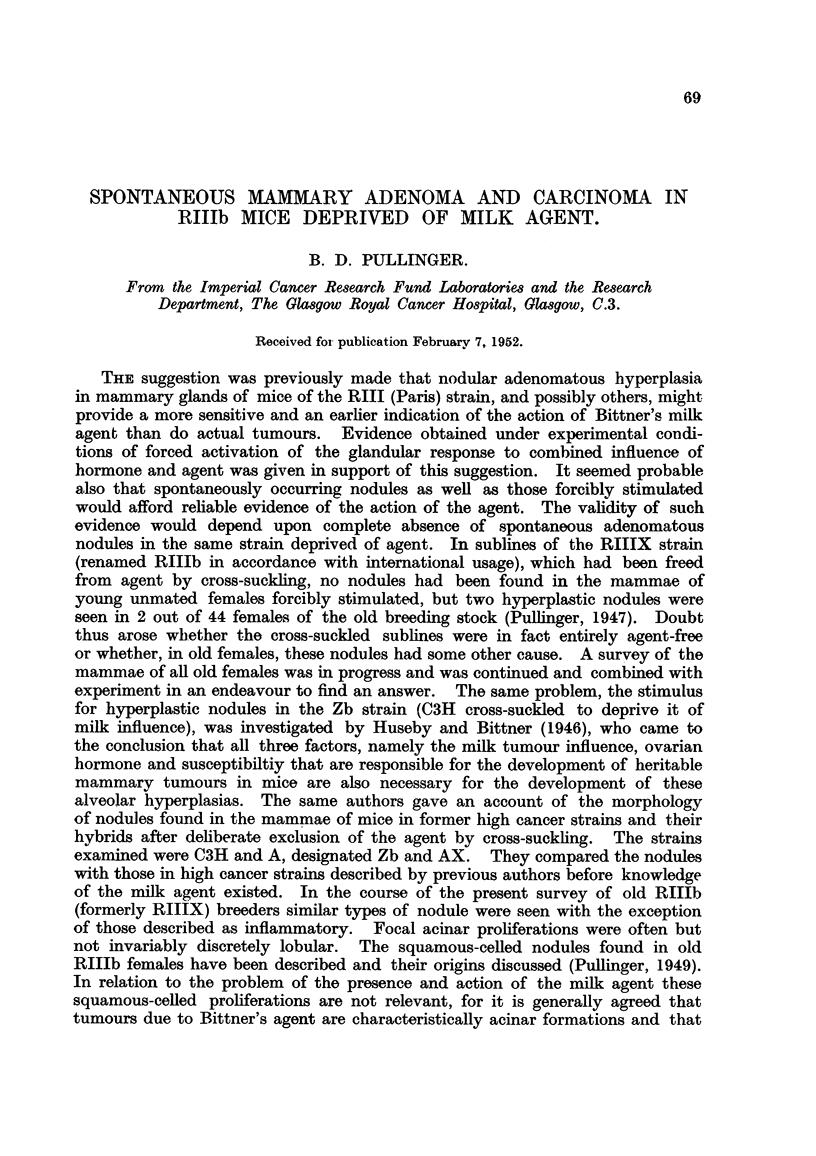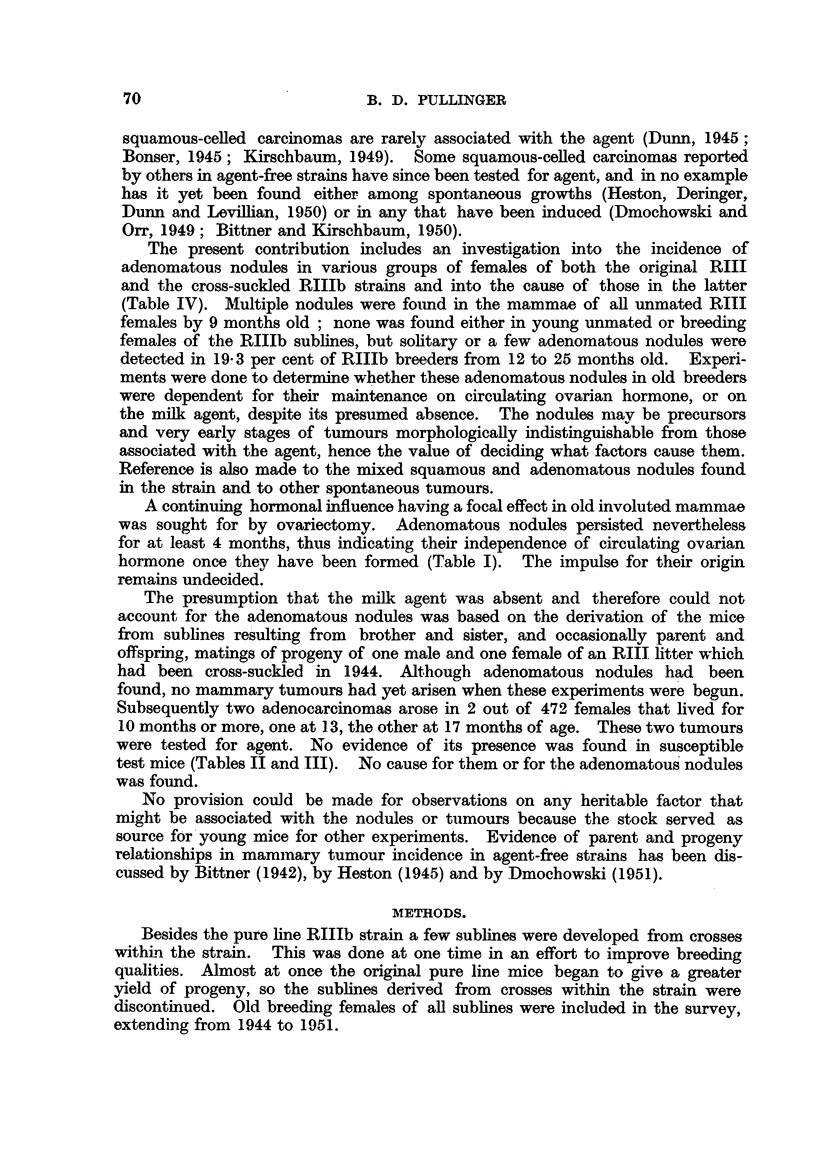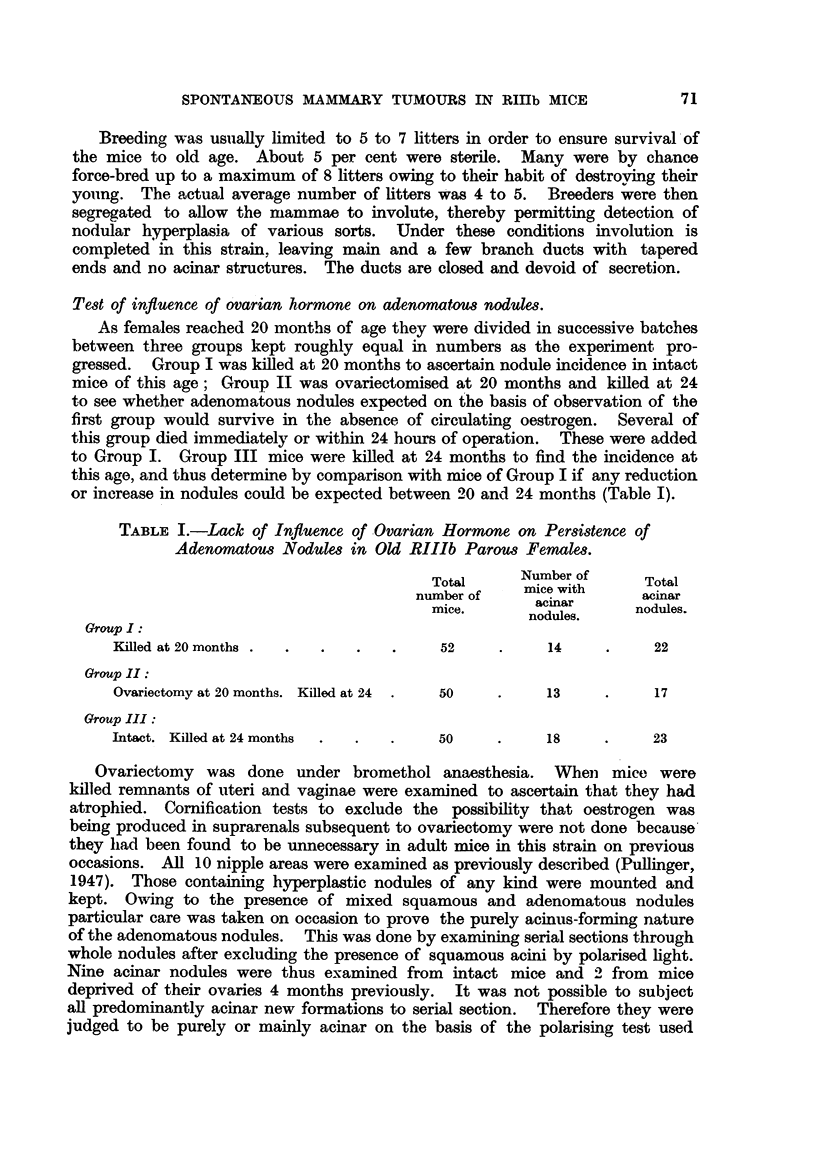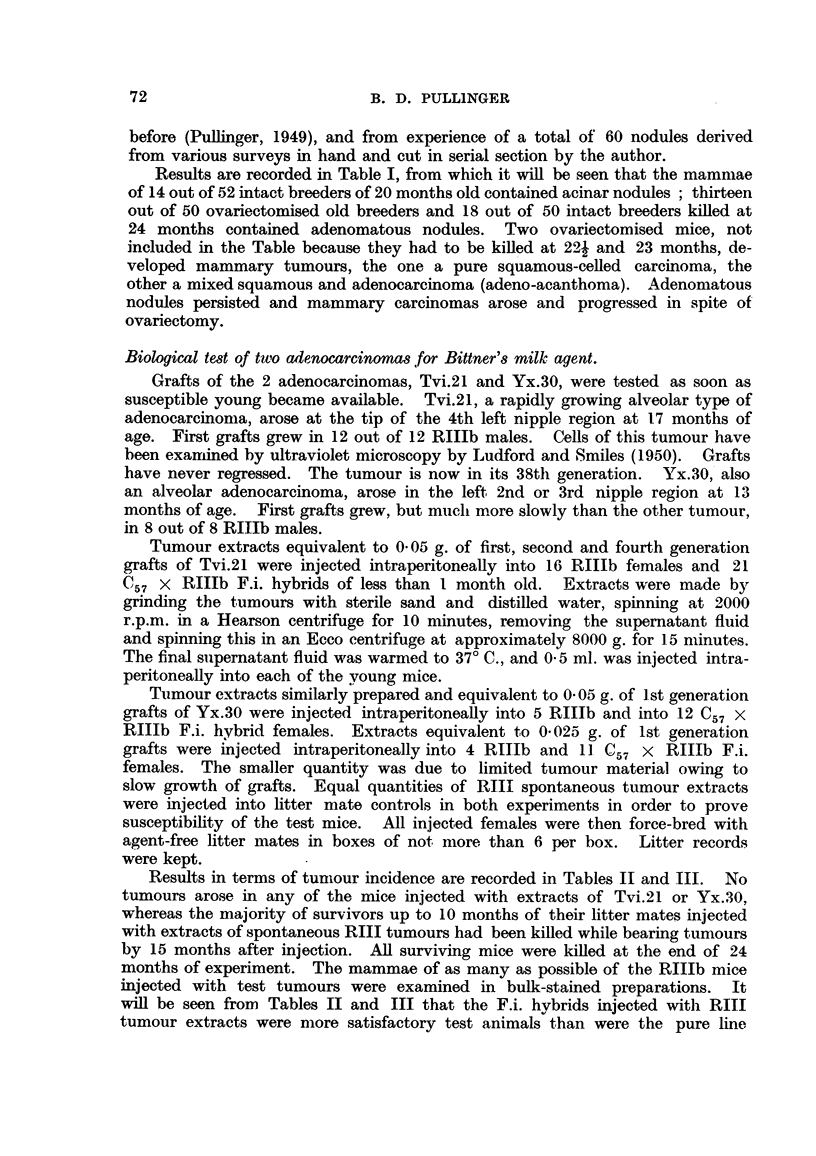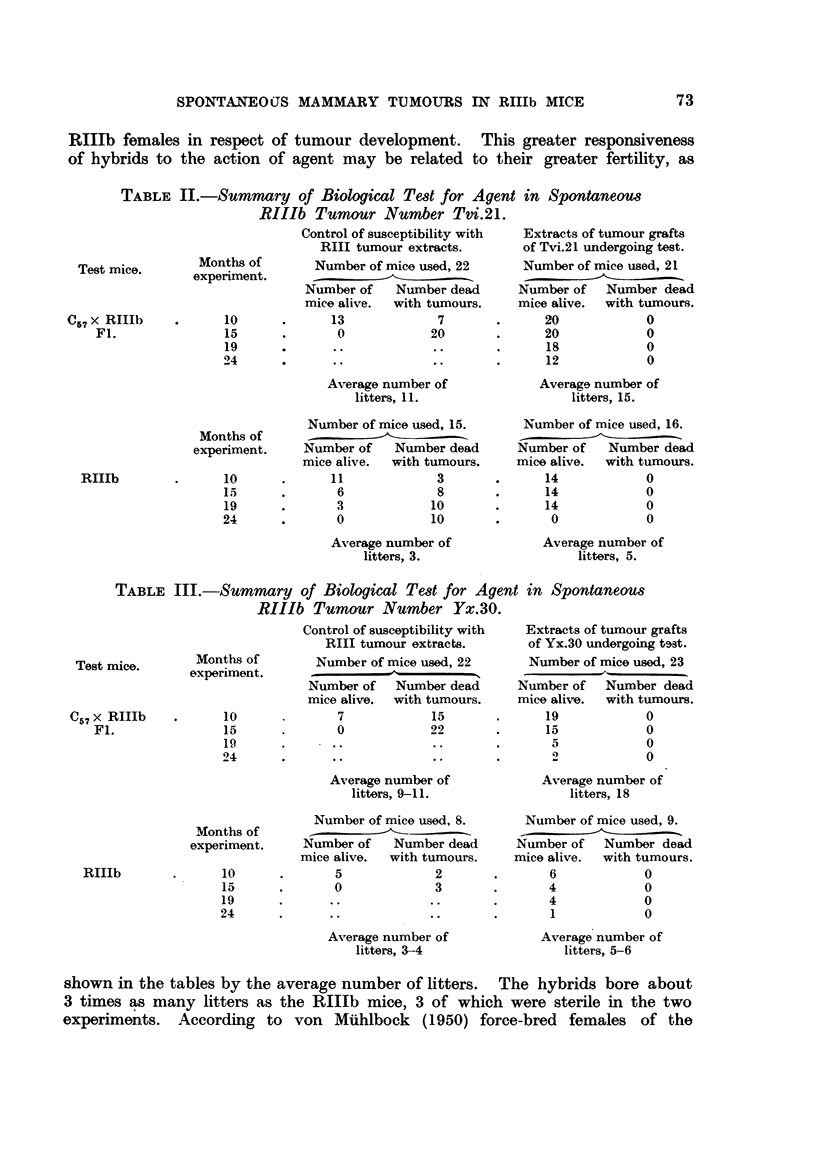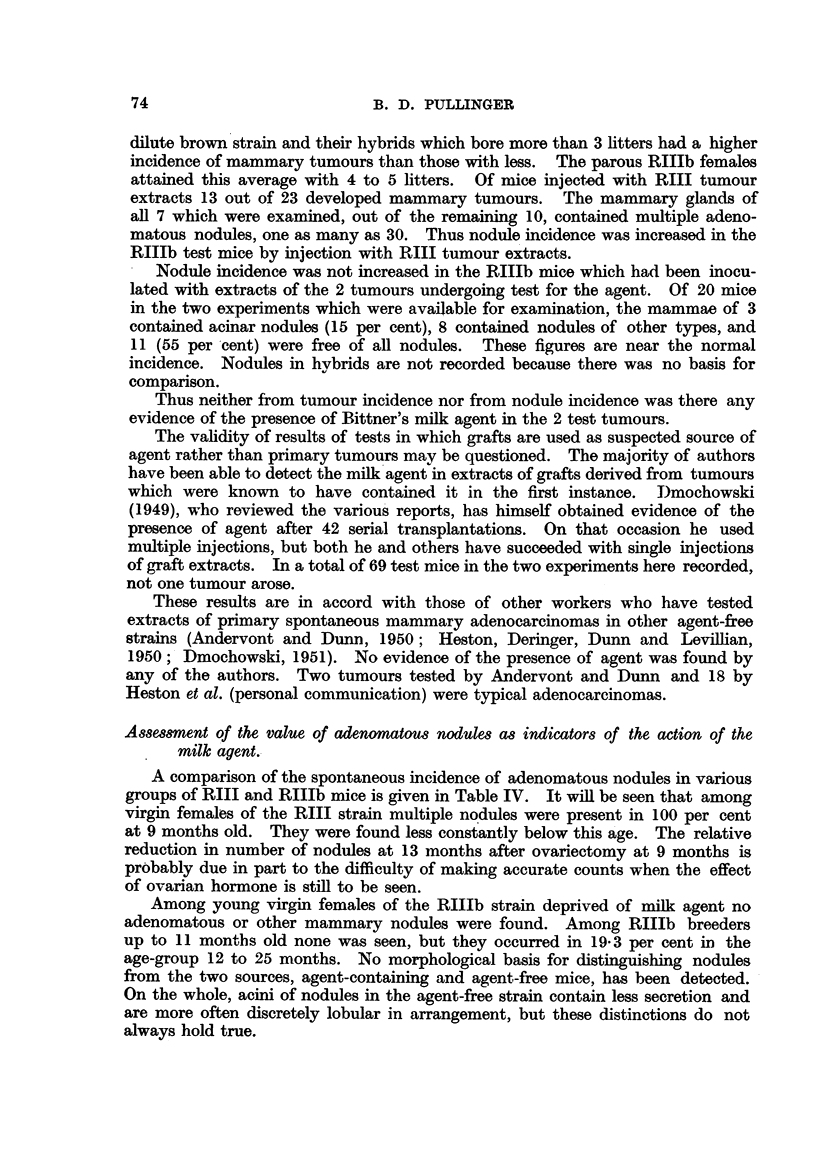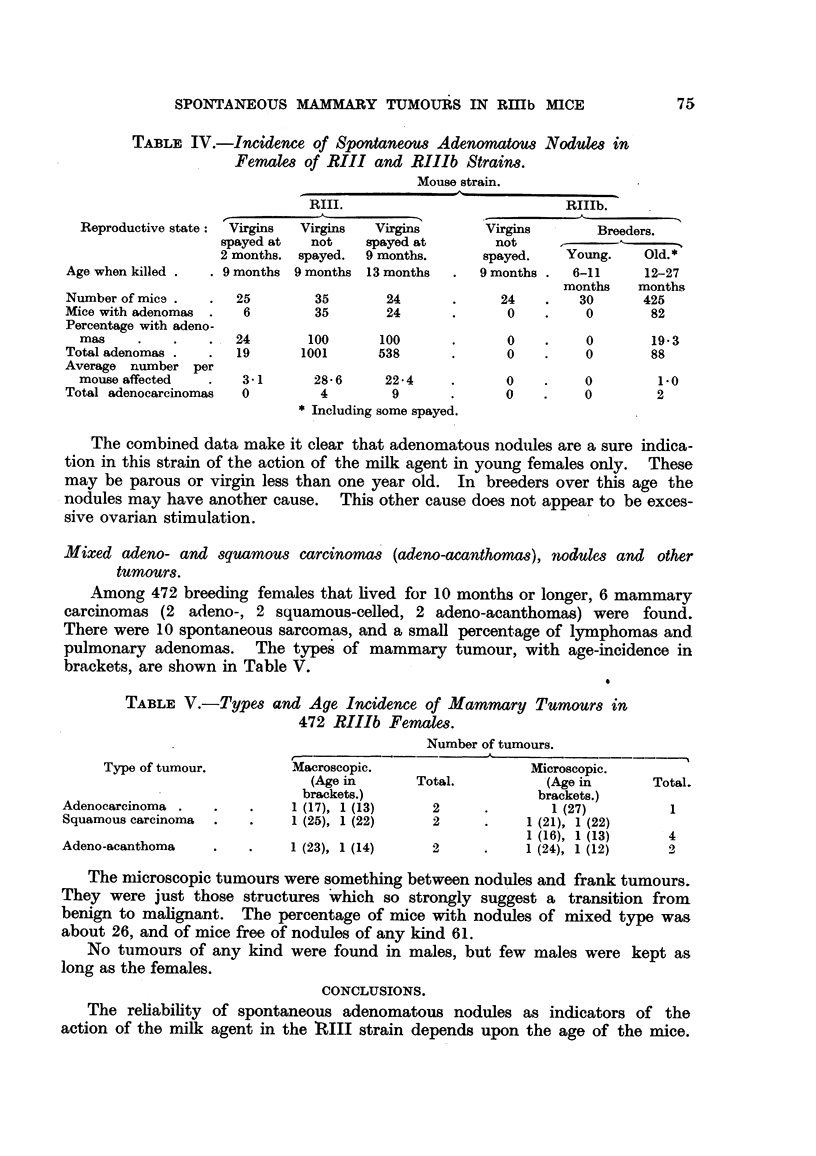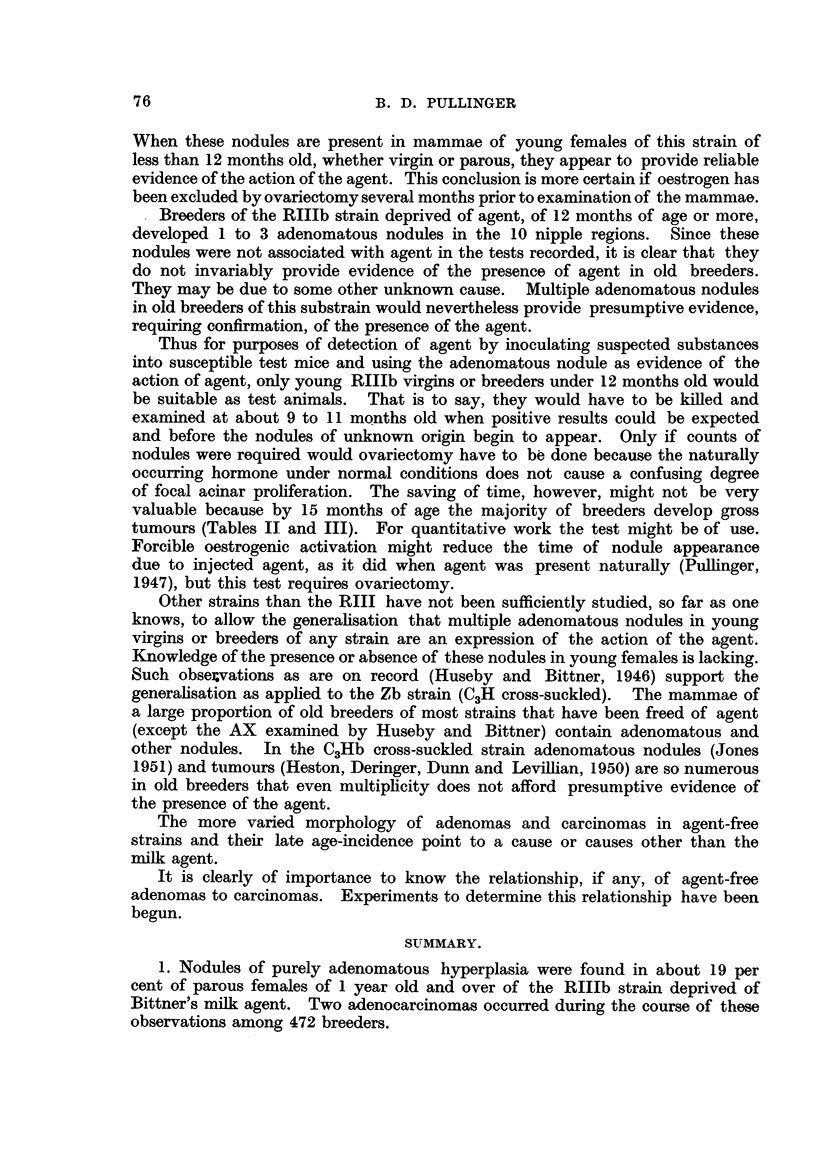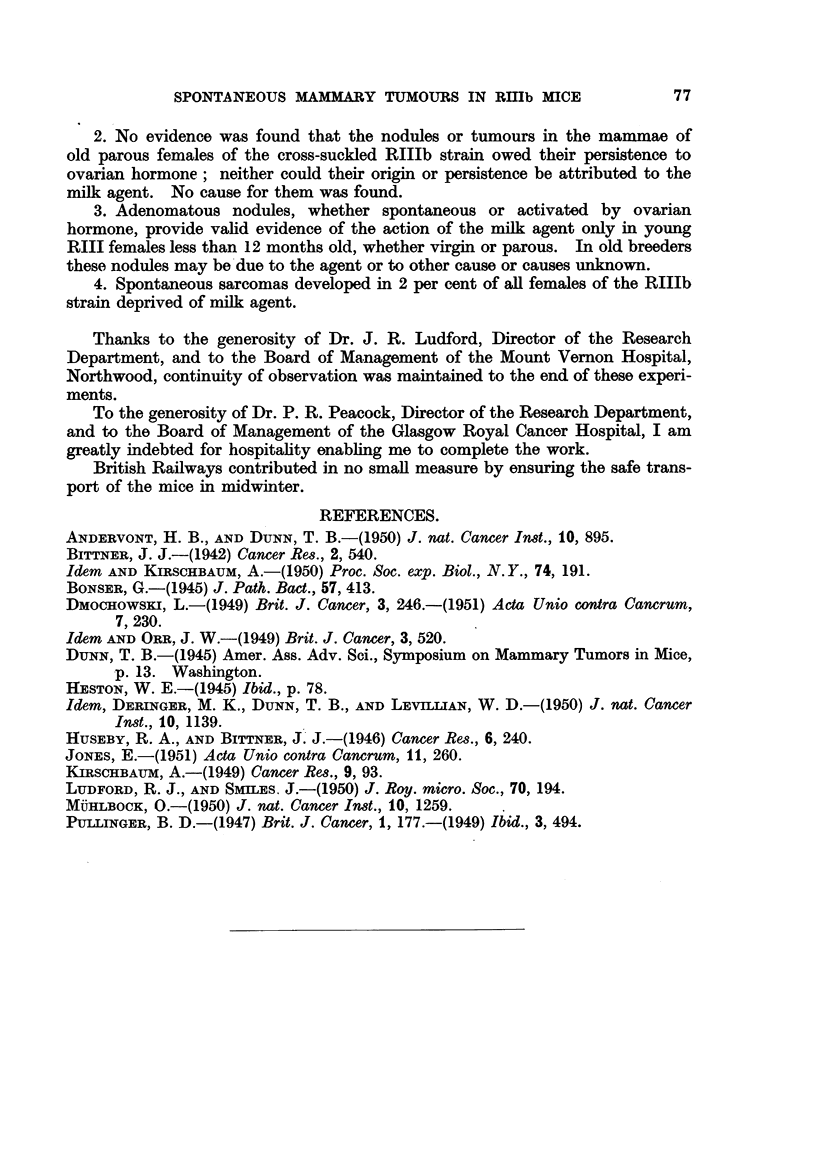# Spontaneous Mammary Adenoma and Carcinoma in RIIIb Mice Deprived of Milk Agent

**DOI:** 10.1038/bjc.1952.6

**Published:** 1952-03

**Authors:** B. D. Pullinger


### SPONTANEOUS MAMMARY ADENOMA AND CARCINOMA IN RIIIb MICE DEPRIVED OF MILK AGENT.

B. D. PULLINGER.

*From the Imperial Cancer Research Fund Laboratories and the Research Department, The Glasgow Royal Cancer Hospital, Glasgow, C.3.*

Received for publication February 7, 1952.

THE suggestion was previously made that nodular adenomatous hyperplasia in mammary glands of mice of the RIII (Paris) strain, and possibly others, might provide a more sensitive and an earlier indication of the action of Bittner's milk agent than do actual tumours. Evidence obtained under experimental conditions of forced activation of the glandular response to combined influence of hormone and agent was given in support of this suggestion. It seemed probable also that spontaneously occurring nodules as well as those forcibly stimulated would afford reliable evidence of the action of the agent. The validity of such evidence would depend upon complete absence of spontaneous adenomatous nodules in the same strain deprived of agent. In sublines of the RIIIX strain (renamed RIIIb in accordance with international usage), which had been freed from agent by cross-suckling, no nodules had been found in the mammae of young unmated females forcibly stimulated, but two hyperplastic nodules were seen in 2 out of 44 females of the old breeding stock (Pullinger, 1947). Doubt thus arose whether the cross-suckled sublines were in fact entirely agent-free or whether, in old females, these nodules had some other cause. A survey of the mammae of all old females was in progress and was continued and combined with experiment in an endeavour to find an answer. The same problem, the stimulus for hyperplastic nodules in the Zb strain (C3H cross-suckled to deprive it of milk influence), was investigated by Huseby and Bittner (1946), who came to the conclusion that all three factors, namely the milk tumour influence, ovarian hormone and susceptibiltiy that are responsible for the development of heritable mammary tumours in mice are also necessary for the development of these alveolar hyperplasias. The same authors gave an account of the morphology of nodules found in the mammae of mice in former high cancer strains and their hybrids after deliberate exclusion of the agent by cross-suckling. The strains examined were C3H and A, designated Zb and AX. They compared the nodules with those in high cancer strains described by previous authors before knowledge of the milk agent existed. In the course of the present survey of old RIIIb (formerly RIIIX) breeders similar types of nodule were seen with the exception of those described as inflammatory. Focal acinar proliferations were often but not invariably discretely lobular. The squamous-celled nodules found in old RIIIb females have been described and their origins discussed (Pullinger, 1949). In relation to the problem of the presence and action of the milk agent these squamous-celled proliferations are not relevant, for it is generally agreed that tumours due to Bittner's agent are characteristically acinar formations and that squamous-celled carcinomas are rarely associated with the agent (Dunn, 1945 ; Bonser, 1945 ; Kirschbaum, 1949). Some squamous-celled carcinomas reported by others in agent-free strains have since been tested for agent, and in no example has it yet been found either among spontaneous growths (Heston, Deringer, Dunn and Levillian, 1950) or in any that have been induced (Dmochowski and Orr, 1949 ; Bittner and Kirschbaum, 1950).

The present contribution includes an investigation into the incidence of adenomatous nodules in various groups of females of both the original RIII and the cross-suckled RIIIb strains and into the cause of those in the latter (Table IV). Multiple nodules were found in the mammae of all unmated RIII females by 9 months old ; none was found either in young unmated or breeding females of the RIIIb sublines, but solitary or a few adenomatous nodules were detected in 19·3 per cent of RIIIb breeders from 12 to 25 months old. Experiments were done to determine whether these adenomatous nodules in old breeders were dependent for their maintenance on circulating ovarian hormone, or on the milk agent, despite its presumed absence. The nodules may be precursors and very early stages of tumours morphologically indistinguishable from those associated with the agent, hence the value of deciding what factors cause them. Reference is also made to the mixed squamous and adenomatous nodules found in the strain and to other spontaneous tumours.

A continuing hormonal influence having a focal effect in old involuted mammae was sought for by ovariectomy. Adenomatous nodules persisted nevertheless for at least 4 months, thus indicating their independence of circulating ovarian hormone once they have been formed (Table I). The impulse for their origin remains undecided.

The presumption that the milk agent was absent and therefore could not account for the adenomatous nodules was based on the derivation of the mice from sublines resulting from brother and sister, and occasionally parent and offspring, matings of progeny of one male and one female of an RIII litter which had been cross-suckled in 1944. Although adenomatous nodules had been found, no mammary tumours had yet arisen when these experiments were begun. Subsequently two adenocarcinomas arose in 2 out of 472 females that lived for 10 months or more, one at 13, the other at 17 months of age. These two tumours were tested for agent. No evidence of its presence was found in susceptible test mice (Tables II and III). No cause for them or for the adenomatous nodules was found.

No provision could be made for observations on any heritable factor that might be associated with the nodules or tumours because the stock served as source for young mice for other experiments. Evidence of parent and progeny relationships in mammary tumour incidence in agent-free strains has been discussed by Bittner (1942), by Heston (1945) and by Dmochowski (1951).

### METHODS.

Besides the pure line RIIIb strain a few sublines were developed from crosses within the strain. This was done at one time in an effort to improve breeding qualities. Almost at once the original pure line mice began to give a greater yield of progeny, so the sublines derived from crosses within the strain were discontinued. Old breeding females of all sublines were included in the survey, extending from 1944 to 1951.

Breeding was usually limited to 5 to 7 litters in order to ensure survival of the mice to old age. About 5 per cent were sterile. Many were by chance force-bred up to a maximum of 8 litters owing to their habit of destroying their young. The actual average number of litters was 4 to 5. Breeders were then segregated to allow the mammae to involute, thereby permitting detection of nodular hyperplasia of various sorts. Under these conditions involution is completed in this strain, leaving main and a few branch ducts with tapered ends and no acinar structures. The ducts are closed and devoid of secretion.

*Test of influence of ovarian hormone on adenomatous nodules.*

As females reached 20 months of age they were divided in successive batches between three groups kept roughly equal in numbers as the experiment progressed. Group I was killed at 20 months to ascertain nodule incidence in intact mice of this age ; Group II was ovariectomised at 20 months and killed at 24 to see whether adenomatous nodules expected on the basis of observation of the first group would survive in the absence of circulating oestrogen. Several of this group died immediately or within 24 hours of operation. These were added to Group I. Group III mice were killed at 24 months to find the incidence at this age, and thus determine by comparison with mice of Group I if any reduction or increase in nodules could be expected between 20 and 24 months (Table I).

TABLE I.—*Lack of Influence of Ovarian Hormone on Persistence of Adenomatous Nodules in Old RIIIb Parous Females.*

| | Total number of mice. | Number of mice with acinar nodules. | Total acinar nodules. |
|---|---|---|---|
| *Group I :* | | | |
| Killed at 20 months | 52 | 14 | 22 |
| *Group II :* | | | |
| Ovariectomy at 20 months. Killed at 24 | 50 | 13 | 17 |
| *Group III :* | | | |
| Intact. Killed at 24 months | 50 | 18 | 23 |

Ovariectomy was done under bromethol anaesthesia. When mice were killed remnants of uteri and vaginae were examined to ascertain that they had atrophied. Cornification tests to exclude the possibility that oestrogen was being produced in suprarenals subsequent to ovariectomy were not done because they had been found to be unnecessary in adult mice in this strain on previous occasions. All 10 nipple areas were examined as previously described (Pullinger, 1947). Those containing hyperplastic nodules of any kind were mounted and kept. Owing to the presence of mixed squamous and adenomatous nodules particular care was taken on occasion to prove the purely acinus-forming nature of the adenomatous nodules. This was done by examining serial sections through whole nodules after excluding the presence of squamous acini by polarised light. Nine acinar nodules were thus examined from intact mice and 2 from mice deprived of their ovaries 4 months previously. It was not possible to subject all predominantly acinar new formations to serial section. Therefore they were judged to be purely or mainly acinar on the basis of the polarising test used before (Pullinger, 1949), and from experience of a total of 60 nodules derived from various surveys in hand and cut in serial section by the author.

Results are recorded in Table I, from which it will be seen that the mammae of 14 out of 52 intact breeders of 20 months old contained acinar nodules ; thirteen out of 50 ovariectomised old breeders and 18 out of 50 intact breeders killed at 24 months contained adenomatous nodules. Two ovariectomised mice, not included in the Table because they had to be killed at 22½ and 23 months, developed mammary tumours, the one a pure squamous-celled carcinoma, the other a mixed squamous and adenocarcinoma (adeno-acanthoma). Adenomatous nodules persisted and mammary carcinomas arose and progressed in spite of ovariectomy.

*Biological test of two adenocarcinomas for Bittner's milk agent.*

Grafts of the 2 adenocarcinomas, Tvi.21 and Yx.30, were tested as soon as susceptible young became available. Tvi.21, a rapidly growing alveolar type of adenocarcinoma, arose at the tip of the 4th left nipple region at 17 months of age. First grafts grew in 12 out of 12 RIIIb males. Cells of this tumour have been examined by ultraviolet microscopy by Ludford and Smiles (1950). Grafts have never regressed. The tumour is now in its 38th generation. Yx.30, also an alveolar adenocarcinoma, arose in the left 2nd or 3rd nipple region at 13 months of age. First grafts grew, but much more slowly than the other tumour, in 8 out of 8 RIIIb males.

Tumour extracts equivalent to 0·05 g. of first, second and fourth generation grafts of Tvi.21 were injected intraperitoneally into 16 RIIIb females and 21 $C_{57}$ × RIIIb F.i. hybrids of less than 1 month old. Extracts were made by grinding the tumours with sterile sand and distilled water, spinning at 2000 r.p.m. in a Hearson centrifuge for 10 minutes, removing the supernatant fluid and spinning this in an Ecco centrifuge at approximately 8000 g. for 15 minutes. The final supernatant fluid was warmed to 37° C., and 0·5 ml. was injected intraperitoneally into each of the young mice.

Tumour extracts similarly prepared and equivalent to 0·05 g. of 1st generation grafts of Yx.30 were injected intraperitoneally into 5 RIIIb and into 12 $C_{57}$ × RIIIb F.i. hybrid females. Extracts equivalent to 0·025 g. of 1st generation grafts were injected intraperitoneally into 4 RIIIb and 11 $C_{57}$ × RIIIb F.i. females. The smaller quantity was due to limited tumour material owing to slow growth of grafts. Equal quantities of RIII spontaneous tumour extracts were injected into litter mate controls in both experiments in order to prove susceptibility of the test mice. All injected females were then force-bred with agent-free litter mates in boxes of not more than 6 per box. Litter records were kept.

Results in terms of tumour incidence are recorded in Tables II and III. No tumours arose in any of the mice injected with extracts of Tvi.21 or Yx.30, whereas the majority of survivors up to 10 months of their litter mates injected with extracts of spontaneous RIII tumours had been killed while bearing tumours by 15 months after injection. All surviving mice were killed at the end of 24 months of experiment. The mammae of as many as possible of the RIIIb mice injected with test tumours were examined in bulk-stained preparations. It will be seen from Tables II and III that the F.i. hybrids injected with RIII tumour extracts were more satisfactory test animals than were the pure line

RIIIb females in respect of tumour development. This greater responsiveness of hybrids to the action of agent may be related to their greater fertility, as

TABLE II.—*Summary of Biological Test for Agent in Spontaneous RIIIb Tumour Number Tvi.*21.

| Test mice. | Months of experiment. | Control of susceptibility with RIII tumour extracts. Number of mice used, 22: Number of mice alive. | Number dead with tumours. | Extracts of tumour grafts of Tvi.21 undergoing test. Number of mice used, 21: Number of mice alive. | Number dead with tumours. |
|---|---|---|---|---|---|
| $C_{57}$ × RIIIb F1. | 10 | 13 | 7 | 20 | 0 |
| | 15 | 0 | 20 | 20 | 0 |
| | 19 | .. | .. | 18 | 0 |
| | 24 | .. | .. | 12 | 0 |
| | | Average number of litters, 11. | | Average number of litters, 15. | |
| | Months of experiment. | Number of mice used, 15. Number of mice alive. | Number dead with tumours. | Number of mice used, 16. Number of mice alive. | Number dead with tumours. |
| RIIIb | 10 | 11 | 3 | 14 | 0 |
| | 15 | 6 | 8 | 14 | 0 |
| | 19 | 3 | 10 | 14 | 0 |
| | 24 | 0 | 10 | 0 | 0 |
| | | Average number of litters, 3. | | Average number of litters, 5. | |

TABLE III.—*Summary of Biological Test for Agent in Spontaneous RIIIb Tumour Number Yx.*30.

| Test mice. | Months of experiment. | Control of susceptibility with RIII tumour extracts. Number of mice used, 22: Number of mice alive. | Number dead with tumours. | Extracts of tumour grafts of Yx.30 undergoing test. Number of mice used, 23: Number of mice alive. | Number dead with tumours. |
|---|---|---|---|---|---|
| $C_{57}$ × RIIIb F1. | 10 | 7 | 15 | 19 | 0 |
| | 15 | 0 | 22 | 15 | 0 |
| | 19 | .. | .. | 5 | 0 |
| | 24 | .. | .. | 2 | 0 |
| | | Average number of litters, 9–11. | | Average number of litters, 18 | |
| | Months of experiment. | Number of mice used, 8. Number of mice alive. | Number dead with tumours. | Number of mice used, 9. Number of mice alive. | Number dead with tumours. |
| RIIIb | 10 | 5 | 2 | 6 | 0 |
| | 15 | 0 | 3 | 4 | 0 |
| | 19 | .. | .. | 4 | 0 |
| | 24 | .. | .. | 1 | 0 |
| | | Average number of litters, 3–4 | | Average number of litters, 5–6 | |

shown in the tables by the average number of litters. The hybrids bore about 3 times as many litters as the RIIIb mice, 3 of which were sterile in the two experiments. According to von Mühlbock (1950) force-bred females of the dilute brown strain and their hybrids which bore more than 3 litters had a higher incidence of mammary tumours than those with less. The parous RIIIb females attained this average with 4 to 5 litters. Of mice injected with RIII tumour extracts 13 out of 23 developed mammary tumours. The mammary glands of all 7 which were examined, out of the remaining 10, contained multiple adenomatous nodules, one as many as 30. Thus nodule incidence was increased in the RIIIb test mice by injection with RIII tumour extracts.

Nodule incidence was not increased in the RIIIb mice which had been inoculated with extracts of the 2 tumours undergoing test for the agent. Of 20 mice in the two experiments which were available for examination, the mammae of 3 contained acinar nodules (15 per cent), 8 contained nodules of other types, and 11 (55 per cent) were free of all nodules. These figures are near the normal incidence. Nodules in hybrids are not recorded because there was no basis for comparison.

Thus neither from tumour incidence nor from nodule incidence was there any evidence of the presence of Bittner's milk agent in the 2 test tumours.

The validity of results of tests in which grafts are used as suspected source of agent rather than primary tumours may be questioned. The majority of authors have been able to detect the milk agent in extracts of grafts derived from tumours which were known to have contained it in the first instance. Dmochowski (1949), who reviewed the various reports, has himself obtained evidence of the presence of agent after 42 serial transplantations. On that occasion he used multiple injections, but both he and others have succeeded with single injections of graft extracts. In a total of 69 test mice in the two experiments here recorded, not one tumour arose.

These results are in accord with those of other workers who have tested extracts of primary spontaneous mammary adenocarcinomas in other agent-free strains (Andervont and Dunn, 1950; Heston, Deringer, Dunn and Levillian, 1950; Dmochowski, 1951). No evidence of the presence of agent was found by any of the authors. Two tumours tested by Andervont and Dunn and 18 by Heston *et al.* (personal communication) were typical adenocarcinomas.

*Assessment of the value of adenomatous nodules as indicators of the action of the milk agent.*

A comparison of the spontaneous incidence of adenomatous nodules in various groups of RIII and RIIIb mice is given in Table IV. It will be seen that among virgin females of the RIII strain multiple nodules were present in 100 per cent at 9 months old. They were found less constantly below this age. The relative reduction in number of nodules at 13 months after ovariectomy at 9 months is probably due in part to the difficulty of making accurate counts when the effect of ovarian hormone is still to be seen.

Among young virgin females of the RIIIb strain deprived of milk agent no adenomatous or other mammary nodules were found. Among RIIIb breeders up to 11 months old none was seen, but they occurred in 19·3 per cent in the age-group 12 to 25 months. No morphological basis for distinguishing nodules from the two sources, agent-containing and agent-free mice, has been detected. On the whole, acini of nodules in the agent-free strain contain less secretion and are more often discretely lobular in arrangement, but these distinctions do not always hold true.

TABLE IV.—*Incidence of Spontaneous Adenomatous Nodules in Females of RIII and RIIIb Strains.*

| | Mouse strain. | | | | | |
|---|---|---|---|---|---|---|
| | RIII. | | | RIIIb. | | |
| | | | | | Breeders. | |
| Reproductive state : | Virgins spayed at 2 months. | Virgins not spayed. | Virgins spayed at 9 months. | Virgins not spayed. | Young. | Old.* |
| Age when killed | 9 months | 9 months | 13 months | 9 months | 6–11 months | 12–27 months |
| Number of mice | 25 | 35 | 24 | 24 | 30 | 425 |
| Mice with adenomas | 6 | 35 | 24 | 0 | 0 | 82 |
| Percentage with adenomas | 24 | 100 | 100 | 0 | 0 | 19·3 |
| Total adenomas | 19 | 1001 | 538 | 0 | 0 | 88 |
| Average number per mouse affected | 3·1 | 28·6 | 22·4 | 0 | 0 | 1·0 |
| Total adenocarcinomas | 0 | 4 | 9 | 0 | 0 | 2 |

* Including some spayed.

The combined data make it clear that adenomatous nodules are a sure indication in this strain of the action of the milk agent in young females only. These may be parous or virgin less than one year old. In breeders over this age the nodules may have another cause. This other cause does not appear to be excessive ovarian stimulation.

*Mixed adeno- and squamous carcinomas (adeno-acanthomas), nodules and other tumours.*

Among 472 breeding females that lived for 10 months or longer, 6 mammary carcinomas (2 adeno-, 2 squamous-celled, 2 adeno-acanthomas) were found. There were 10 spontaneous sarcomas, and a small percentage of lymphomas and pulmonary adenomas. The types of mammary tumour, with age-incidence in brackets, are shown in Table V.

TABLE V.—*Types and Age Incidence of Mammary Tumours in 472 RIIIb Females.*

| | Number of tumours. | | | |
|---|---|---|---|---|
| Type of tumour. | Macroscopic. (Age in brackets.) | Total. | Microscopic. (Age in brackets.) | Total. |
| Adenocarcinoma | 1 (17), 1 (13) | 2 | 1 (27) | 1 |
| Squamous carcinoma | 1 (25), 1 (22) | 2 | 1 (21), 1 (22)<br>1 (16), 1 (13) | 4 |
| Adeno-acanthoma | 1 (23), 1 (14) | 2 | 1 (24), 1 (12) | 2 |

The microscopic tumours were something between nodules and frank tumours. They were just those structures which so strongly suggest a transition from benign to malignant. The percentage of mice with nodules of mixed type was about 26, and of mice free of nodules of any kind 61.

No tumours of any kind were found in males, but few males were kept as long as the females.

### CONCLUSIONS.

The reliability of spontaneous adenomatous nodules as indicators of the action of the milk agent in the RIII strain depends upon the age of the mice.

When these nodules are present in mammae of young females of this strain of less than 12 months old, whether virgin or parous, they appear to provide reliable evidence of the action of the agent. This conclusion is more certain if oestrogen has been excluded by ovariectomy several months prior to examination of the mammae.

Breeders of the RIIIb strain deprived of agent, of 12 months of age or more, developed 1 to 3 adenomatous nodules in the 10 nipple regions. Since these nodules were not associated with agent in the tests recorded, it is clear that they do not invariably provide evidence of the presence of agent in old breeders. They may be due to some other unknown cause. Multiple adenomatous nodules in old breeders of this substrain would nevertheless provide presumptive evidence, requiring confirmation, of the presence of the agent.

Thus for purposes of detection of agent by inoculating suspected substances into susceptible test mice and using the adenomatous nodule as evidence of the action of agent, only young RIIIb virgins or breeders under 12 months old would be suitable as test animals. That is to say, they would have to be killed and examined at about 9 to 11 months old when positive results could be expected and before the nodules of unknown origin begin to appear. Only if counts of nodules were required would ovariectomy have to be done because the naturally occurring hormone under normal conditions does not cause a confusing degree of focal acinar proliferation. The saving of time, however, might not be very valuable because by 15 months of age the majority of breeders develop gross tumours (Tables II and III). For quantitative work the test might be of use. Forcible oestrogenic activation might reduce the time of nodule appearance due to injected agent, as it did when agent was present naturally (Pullinger, 1947), but this test requires ovariectomy.

Other strains than the RIII have not been sufficiently studied, so far as one knows, to allow the generalisation that multiple adenomatous nodules in young virgins or breeders of any strain are an expression of the action of the agent. Knowledge of the presence or absence of these nodules in young females is lacking. Such observations as are on record (Huseby and Bittner, 1946) support the generalisation as applied to the Zb strain ($C_3$H cross-suckled). The mammae of a large proportion of old breeders of most strains that have been freed of agent (except the AX examined by Huseby and Bittner) contain adenomatous and other nodules. In the $C_3$Hb cross-suckled strain adenomatous nodules (Jones 1951) and tumours (Heston, Deringer, Dunn and Levillian, 1950) are so numerous in old breeders that even multiplicity does not afford presumptive evidence of the presence of the agent.

The more varied morphology of adenomas and carcinomas in agent-free strains and their late age-incidence point to a cause or causes other than the milk agent.

It is clearly of importance to know the relationship, if any, of agent-free adenomas to carcinomas. Experiments to determine this relationship have been begun.

### SUMMARY.

1. Nodules of purely adenomatous hyperplasia were found in about 19 per cent of parous females of 1 year old and over of the RIIIb strain deprived of Bittner's milk agent. Two adenocarcinomas occurred during the course of these observations among 472 breeders.

2. No evidence was found that the nodules or tumours in the mammae of old parous females of the cross-suckled RIIIb strain owed their persistence to ovarian hormone ; neither could their origin or persistence be attributed to the milk agent. No cause for them was found.

3. Adenomatous nodules, whether spontaneous or activated by ovarian hormone, provide valid evidence of the action of the milk agent only in young RIII females less than 12 months old, whether virgin or parous. In old breeders these nodules may be due to the agent or to other cause or causes unknown.

4. Spontaneous sarcomas developed in 2 per cent of all females of the RIIIb strain deprived of milk agent.

Thanks to the generosity of Dr. J. R. Ludford, Director of the Research Department, and to the Board of Management of the Mount Vernon Hospital, Northwood, continuity of observation was maintained to the end of these experiments.

To the generosity of Dr. P. R. Peacock, Director of the Research Department, and to the Board of Management of the Glasgow Royal Cancer Hospital, I am greatly indebted for hospitality enabling me to complete the work.

British Railways contributed in no small measure by ensuring the safe transport of the mice in midwinter.

### REFERENCES.

ANDERVONT, H. B., AND DUNN, T. B.—(1950) *J. nat. Cancer Inst.*, **10**, 895.
BITTNER, J. J.—(1942) *Cancer Res.*, **2**, 540.
*Idem* AND KIRSCHBAUM, A.—(1950) *Proc. Soc. exp. Biol., N.Y.*, **74**, 191.
BONSER, G.—(1945) *J. Path. Bact.*, **57**, 413.
DMOCHOWSKI, L.—(1949) *Brit. J. Cancer*, **3**, 246.—(1951) *Acta Unio contra Cancrum*, **7**, 230.
*Idem* AND ORR, J. W.—(1949) *Brit. J. Cancer*, **3**, 520.
DUNN, T. B.—(1945) Amer. Ass. Adv. Sci., Symposium on Mammary Tumors in Mice, p. 13. Washington.
HESTON, W. E.—(1945) *Ibid.*, p. 78.
*Idem*, DERINGER, M. K., DUNN, T. B., AND LEVILLIAN, W. D.—(1950) *J. nat. Cancer Inst.*, **10**, 1139.
HUSEBY, R. A., AND BITTNER, J. J.—(1946) *Cancer Res.*, **6**, 240.
JONES, E.—(1951) *Acta Unio contra Cancrum*, **11**, 260.
KIRSCHBAUM, A.—(1949) *Cancer Res.*, **9**, 93.
LUDFORD, R. J., AND SMILES, J.—(1950) *J. Roy. micro. Soc.*, **70**, 194.
MÜHLBOCK, O.—(1950) *J. nat. Cancer Inst.*, **10**, 1259.
PULLINGER, B. D.—(1947) *Brit. J. Cancer*, **1**, 177.—(1949) *Ibid.*, **3**, 494.